# To adjust or not to adjust? Matching statistical choices to scientific questions in exercise physiology and sport science

**DOI:** 10.3389/fphys.2026.1834628

**Published:** 2026-06-10

**Authors:** Gudrun Schappacher-Tilp, Markus Tilp

**Affiliations:** 1 Department of Engineering, FH JOANNEUM– University of Applied Sciences, Graz, Austria; 2 Department of Human Movement Sciences, Sport and Health, University of Graz, Graz, Austria

**Keywords:** familywise error, multiplicity adjustments, multiplicity correction, Omnibus test, *post hoc* test, statistics

## Abstract

Adjustment for multiple statistical tests has become a default marker of rigor in clinical and health research. We argue that its routine application is often conceptually inappropriate and can obscure meaningful results. The need for multiplicity correction depends not simply on how many tests are performed, but on the inferential question being asked. When the aim is to determine whether any detectable effect exists, an omnibus test is the appropriate inferential tool, and subsequent *post hoc* comparisons serve a descriptive role. When a small number of directed hypotheses are specified *a priori*, routine blanket correction across all conceivable contrasts is not required, provided the hypothesis set is fixed independently of the data. By contrast, when the aim is to identify where an effect is visible across a broader set of candidate contrasts, the problem is one of multiple inference and calls for explicit error control, such as false discovery rate control or, where stricter control is required, familywise error control. A related but distinct issue arises when multiple variables reflect different aspects of the same underlying biological process. Here, simple count-based adjustment penalizes measurement richness without addressing a coherent inferential problem and should be replaced by consideration of the latent structure of the outcome space. We propose a context-dependent framework, operationalized through a small set of guiding questions, that links inferential aims to appropriate statistical procedures across three modes: global detection, directed hypothesis testing, and exploratory localization. This framework supports more transparent and defensible statistical practice.

## Introduction

Applied sport and health science research frequently involves the simultaneous analysis of multiple outcomes, including physiological, biomechanical, and perceptual measures. In this setting, a fixed analytical sequence has become standard practice: an omnibus test followed by multiplicity-corrected *post-hoc* comparisons, routinely expected by reviewers and editors as an indicator of methodological rigor.

However, this sequence is rarely justified in relation to the scientific question being asked. The choice of primary inferential tool and the decision to apply multiplicity correction are typically treated as procedural defaults rather than deliberate responses to the structure of the research problem.

This Perspective argues that the mechanical application of this sequence is often conceptually inappropriate and can obscure meaningful physiological results. Whether correction is warranted, and whether an omnibus test should be performed at all, depends not on the number of tests but on the inferential goal of the analysis.

We propose a context-dependent framework, operationalized as a decision table, that links inferential goals to appropriate statistical procedures across three modes: global detection, directed hypothesis testing, and exploratory localization. This replaces a mechanical testing sequence with principled, question-driven inference.

## The tension in modern sport science statistics

In practice, the combination of an omnibus test followed by multiplicity-corrected *post-hoc* comparisons has become a default analytical ritual rather than a purposeful response to a specific research question. This automaticity is reinforced by widely used statistical software, where *post-hoc* correction options are presented as a standard extension of the omnibus test procedure. As a result, the sequence is applied as a concession to perceived expectations rather than as a deliberate inferential choice.

The software-driven ritual occurs against a backdrop of increasing concern regarding the replicability of sport and exercise science research, where inflated Type I error rates are frequently blamed for diminishing effect sizes in follow-up studies (Murphy et al., 2025). However, the reflexive mandate for stricter false-positive control (Anderson and Post, 2025) ignores a fundamental conceptual mismatch: while these corrections are intended to protect against ‘p-hacking’ in broad, undirected screening, their application in directed or descriptive settings imposes an overly conservative penalty that erases physiologically meaningful results (Berger and Sellke, 1987; Rothman, 1990).

From our perspective, this reflects a broader conflation of statistical ritual with scientific rigor and the ritual in question is not correction alone, but the unexamined assumption that every multi-group analysis should begin with a global null test.

## The decision-theoretic trap in applied research

Classical statistical tests were developed as decision procedures for repetitive situations within the Neyman–Pearson framework (Neyman and Pearson, 1933). Their purpose is to control long-run error rates when the same decision is made repeatedly under similar conditions. In this framework, a Type I error refers to rejecting a true null hypothesis.

Importantly, Type I error characterizes the testing procedure, not individual results, and refers to the rate of false-positive conclusions under repeated application of the procedure when the null hypothesis is true (Neyman and Pearson, 1933). Adjustment for multiple tests follows from this logic: when many tests compete to reject a global null hypothesis, tightening the decision rule limits false-positive conclusions at the family level (Neyman and Pearson, 1933; Rothman, 1990; Bland and Altman, 1995). This logic applies specifically when the research aim is to determine whether any effect exists at all. It includes broad screening analyses, large-scale outcome screening, and repeated testing scenarios where multiple hypotheses compete to detect any signal.

Problems arise when this decision-theoretic logic is applied uncritically to applied sport and health science, where studies are typically unique, context-dependent, and focused on interpretation rather than repeated decision-making (Berger and Sellke, 1987; Goodman, 1999). In our view, this mismatch between theory and application underlies much of the confusion surrounding multiplicity in applied research. When the research question is not global — when it concerns the size or direction of a specific effect rather than the existence of any detectable effect — the decision-theoretic rationale for correction does not apply and invoking it through an omnibus test adds a procedural step without inferential justification.

## Three inferential modes, three different answers

The appropriate inferential tool depends entirely on the scientific question being asked.

If the question is global, that is, whether any systematic differences is detectable at all, an omnibus test is the correct choice. It tests precisely this hypothesis under controlled Type I error, and subsequent *post-hoc* comparisons serve a descriptive role: they characterize the structure of an already-established effect and do not constitute individual confirmatory claims. No multiplicity correction is required (Rothman, 1990; Perneger, 1998).

If the aim is to evaluate a small number of directed, pre-specified contrasts, the omnibus test is the wrong starting point as it answers a question that was not asked. Directed comparisons should be conducted directly, without a preceding omnibus test. Because the hypothesis set is fixed in advance and not influenced by the observed data, routine blanket correction across all conceivable contrasts is not required (Rosenthal et al., 1999; Rubin, 2021).

If the aim is to identify which among a broader set of contrasts reach significance, the analysis constitutes a multiple inference problem: contrasts compete for inclusion in the set of reported findings, and no prior theory singles out any one of them as the expected locus of an effect. This is the setting for which multiplicity correction was developed. It includes broad outcome screening without pre-specified hypotheses, repeated testing across many subsamples without an expectation of heterogeneity, sequential analyses, or genuinely undirected investigations (Neyman and Pearson, 1933; Tukey, 1949; Rothman, 1990; Savitz and Olshan, 1995; Perneger, 1998; Anderson and Post, 2025). Explicit error control is required, for example through false discovery rate control (Benjamini and Hochberg, 1995) or, where stricter guarantees are needed, familywise error control. In this setting too, a preceding omnibus test does not address the actual inferential question and should not be used as a procedural gateway.

This distinction explains a familiar and frustrating pattern in sport and health science: a significant omnibus test followed by *post-hoc* comparisons that are all declared non-significant. This apparent paradox arises because the omnibus test addresses the existence of an effect, whereas *post hoc* comparisons, when subjected to strict multiplicity control, are required to meet a different and more demanding inferential criterion. The issue is therefore not a lack of support for the omnibus result, but a mismatch between inferential targets (Rothman, 1990; Savitz and Olshan, 1995; Perneger, 1998). This issue is not limited to commonly used procedures such as Tukey or Bonferroni. More general procedures, such as the Scheffé test (Scheffe, 1953), resolve the logical inconsistency by controlling the familywise error rate across all possible contrasts, but only by extending the interferential scope to the full set of linear combinations, an effectively infinite set of which many are not of substantive interest. Even scientifically motivated and pre-specified contrasts may therefore fail to reach significance. From our perspective, this reflects a deeper inferential mismatch. Framing *post-hoc* comparisons as a separate multiple-inference problem assumes that a study simultaneously pursues both a global and a contrast-specific inferential goal. While common in practice, we question whether this reflects the scientific question in many exercise physiology applications. If specific comparisons are the primary interest, they should generally define the primary inferential analysis rather than depend on rejection of an omnibus null hypothesis that is not itself the substantive target of inference. The core issue is therefore two-fold: the choice of correction procedure cannot be separated from the choice of primary inferential tool. If the scientific question is global, the omnibus test answers it and *post-hoc* correction is unnecessary. If the question is not global, for example, when the aim is to determine which training modalities improve performance relative to control, or at which time point an adaptation first becomes detectable, the omnibus test should not be performed at all. In such cases, whether correction is needed depends entirely on whether the comparisons are directed or exploratory, and that decision must be made before the data collection.

## Illustrative examples: three questions, three analyses

To illustrate how the inferential mode shapes the appropriate statistical approach, we present three scenarios, each asking a different scientific question about training-induced adaptation.

### Example 1: directed hypothesis testing

VO_2_max was assessed in physically active women before and after a 6-week training period in three groups: control, Moderate-Intensity Continuous Training (MICT), and High-Intensity Interval Training (HIIT). The simulation was designed to reflect realistic variability and effect sizes observed in training studies (data provided in the Supplement).

The scientific questions are directed and pre-specified: does MICT improve VO_2_max relative to control, and does HIIT? These are two pre-specified, theoretically motivated hypotheses and not an undirected search for any difference among groups. The appropriate analysis is to test each contrast directly, without a preceding omnibus test (Rubin, 2021). Because the hypothesis set is fixed independently of the data, routine multiplicity correction is not required.

**Figure 1:**
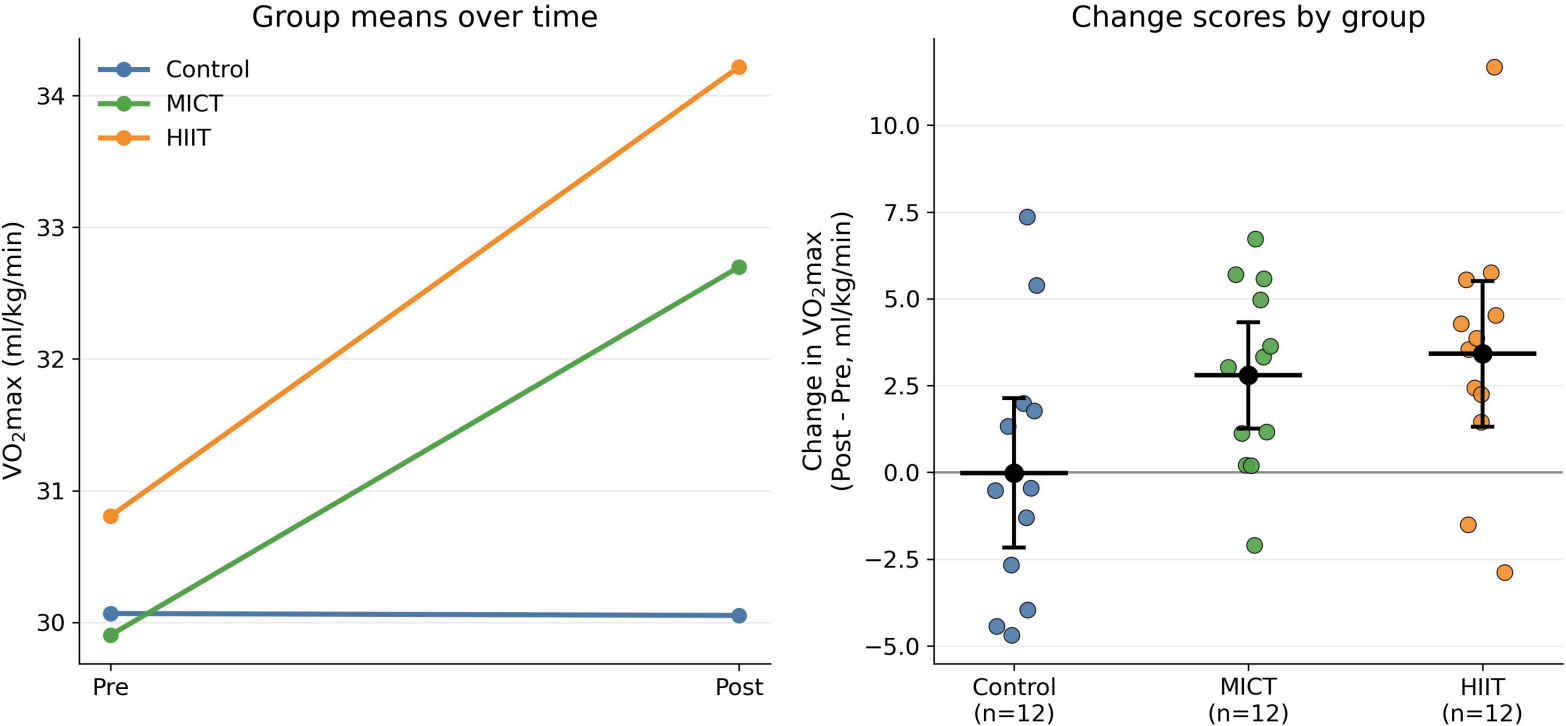
Simulated changes in VO_2_max following Control, Moderate-Intensity Continous Training (MICT), and High-Intensity Intervall Training (HIIT) interventions. Left panel: Group mean VO_2_max values before (Pre) and after (Post) the intervention period. Right panel: Individual change scores (Post − Pre) with group means and variability indicated. All assumptions for repeated-measures ANOVA including normality of residuals and homogeneity of variances were met, ensuring that any failure to detect significance under the mechanical approach cannot be attributed to assumption violations.

What if we apply the mechanical approach instead, i.e. ANOVA followed by corrected *post-hoc* comparisons? The ANOVA tests the global null hypothesis that no differences exist among any groups, which was not the question. Its significant result (F(2,33) = 3.393, p = 0.045) confirms only that some difference exists somewhere, a finding already implied by the study hypotheses. The subsequent *post-hoc* comparisons failed to reach significance under any correction (Bonferroni/Holm: p = 0.106; Tukey: p = 0.051; Scheffé: p = 0.064), despite the HIIT versus control contrast showing a clinically meaningful difference of 3.43 mL·kg^-^¹·min^-^¹ that was significant when tested directly (p = 0.036, **Figure 1**).

The ANOVA added a procedural detour, and the corrections penalized a directed comparison for belonging to a family it does not logically belong to.

### Example 2: exploratory localization

The same HIIT intervention is examined in a single group, with VO_2_max measured at baseline and at weeks 2, 4, and 6. Training-induced improvements over six weeks are well established; the question is not whether an effect exists, but when it first becomes detectable.

This is an exploratory localization problem. The three comparisons, baseline to week 2, baseline to week 4, and baseline to week 6, are not directed by prior theory. No principled reason exists to single out any time point as the expected locus of first detection. The comparisons therefore compete to identify the earliest significant departure from baseline, which is precisely the disjunctive structure that warrants error control (Scheffe, 1953): a false positive at any time point would support an incorrect claim about the onset of adaptation, and that risk accumulates across comparisons.

The appropriate analysis is to test each contrast directly, without a preceding omnibus test. A repeated-measures ANOVA would test whether any differences exist across time points, which is already assumed and therefore uninformative. False discovery rate control is the natural choice for error control, limiting the expected proportion of false positives among significant findings without imposing the more conservative familywise guarantee that would be warranted only if the validity of every individual claim required independent protection (Benjamini and Hochberg, 1995).

The contrast with Example 1 is instructive. In Example 1, correction penalizes directed comparisons for belonging to a family they do not logically belong to. Here, correction is not only appropriate but necessary. In both cases the omnibus test is the wrong starting point, because in neither scenario is the global null the scientific question.

### Example 3: global detection

A sports science consortium evaluates the reproducibility of post-exercise blood lactate measurements across six independent laboratories. All laboratories follow the same standardized protocol but use different equipment and personnel.

The scientific question is explicitly global: is there any systematic laboratory effect at all? The goal is not to identify which laboratories differ or to rank them, but to determine whether the measurement process is consistent across settings. A significant result indicates a reproducibility problem somewhere in the system. A non-significant result supports pooling data across sites. The decision is binary and hinges on a single inferential step.

The omnibus F-test addresses this question directly and completely. If the global null, that all laboratory means are equal, is rejected under controlled Type I error, the conclusion is that laboratory-dependent variation exists. No further inference about specific laboratories is required, because no individual claims about specific sites are of interest. *Post-hoc* comparisons are descriptive, not confirmatory, and require no correction.

What makes the omnibus test appropriate here is not the number of groups but the nature of the question: the global null itself is the scientific objective. This situation is uncommon in exercise physiology and sport science, where most studies aim to evaluate specific interventions or identify where differences occur rather than whether any exist at all. Recognizing this distinction is the first step toward matching the inferential tool to the question.

## The “richness penalty” in multidimensional data

The three inferential modes described above share a common assumption: that the variables under analysis represent distinct targets of inference. A related but structurally different problem arises when multiple variables are not independent discovery attempts but different facets of the same underlying physiological process. Here, the multiplicity question cannot be resolved by identifying the inferential mode. It requires asking whether the variables are conceptually exchangeable or whether they jointly index a single latent construct. Where they do not — where variables are correlated but serve as independent targets of inference rather than facets of a single construct — the inferential mode framework applies. Whether correction is needed depends on whether the comparisons are directed or exploratory, not on the statistical correlation between outcomes.

The problem becomes concrete when adjustment is applied to outcome sets where variables jointly reflect a single underlying process. Correction implies that the interpretation of a given comparison depends on how many other tests happened to be performed. An observed result may therefore be treated as informative in one analysis but dismissed in another simply because additional variables were included.

From an applied perspective, this defies common sense: what the data show about a specific measure does not change merely because other measures were recorded.

This is particularly salient in sport science, where variables such as lactate concentration, heart rate, and perceived exertion, or multiple split times from a single sprint, represent different views of the same underlying physiological or performance process. An elevated lactate concentration does not become uninformative simply because several other related measures were analyzed and adjustment renders the comparison non-significant. Treating correlated measures as independent discovery attempts penalizes researchers for measurement richness rather than addressing a genuine error-control problem. The number of instruments used to observe a phenomenon should not determine whether the phenomenon is considered detectable.

The appropriate response is a different framing of the inferential unit. When variables jointly reflect a single underlying construct, that construct becomes the natural target of inference. Analytical approaches that respect this structure, such as multivariate test statistics or dimension reduction prior to testing, address the actual inferential problem. At minimum, researchers should ask whether outcome variables are conceptually independent before applying count-based correction at all. Practical starting points include pre-specifying one primary outcome per construct, grouping variables by conceptual family before analysis, applying multivariate tests to each family, and reporting all measured variables regardless of significance to reduce the risk of selective interpretation.

## Discussion

Our analysis suggests that the problem is not multiplicity itself but the failure to match the statistical procedure to the inferential question. Researchers should identify which inferential mode their analysis occupies before deciding whether an omnibus test or correction is appropriate.

Correction is warranted when the scientific question is global and undirected. It is not warranted when hypotheses are directed and fixed in advance, when *post-hoc* comparisons describe the structure of an already-established effect, or when correlated variables jointly index a single underlying construct. Applying correction indiscriminately across these situations conflates the appearance of rigor with its substance. Reviewers and editors should encourage explicit justification of the inferential mode rather than requiring adherence to standard correction procedures as a condition of publication.

The framework proposed here reframes the role of the omnibus test. It is not a prerequisite for multiple comparisons but the appropriate inferential tool when and only when the global null is the scientific question. When directed contrasts or exploratory localization are the goal, the omnibus test answers a question that was not asked and should not be performed. This has direct implications for how intervention studies in exercise physiology are designed, analyzed, and reported.

Concerns about false positives and *post hoc* interpretation are valid, but they arise primarily from selective reporting and flexible analytical decisions rather than from the mere presence of multiple tests. These are issues of transparency, not error control (Berger and Sellke, 1987; Goodman, 1999; Murphy et al., 2025). Clear reporting of what was tested, why it was tested, and how results should be interpreted ideally through pre-registration and effect size reporting, allows informed evaluation without reliance on mechanical adjustment and mitigates concerns such as selective reporting or p-hacking (Simmons et al., 2011).

Statistical rigor in sport and health science is achieved by aligning the analytical method with the scientific question and reporting that alignment transparently so that readers can evaluate it (**Table 1**).

**Table 1:** To adjust or not to adjust? a decision framework for the three inferential modes.

Inferential mode	Scientific question	Primary tool	Adjust?	Rationale
Global detection	Are there any differences at all?	Omnibus test (e.g. ANOVA)	Not required	The omnibus test is the error-control mechanism. *Post-hoc* comparisons are purely descriptive and require no further correction.
Directed hypothesis testing	Do these specific, pre-specified contrasts reach significance?	Planned comparisons, direct t-tests	Not required	Hypotheses are fixed independently of the data. Correction penalizes directed inference for a multiple-discovery problem that does not apply.
Exploratory localisation	Where among a broader set of contrasts is an effect detectable?	Direct comparisons with FDR or FWER control	Yes	Contrasts compete to detect any signal. Error control is required to limit false-positive localization claims.
Measurement richness	How is a single underlying construct expressed across correlated indicators?	Multivariate tests, dimension reduction, latent construct modelling	Not required	Correlated variables are not independent discovery attempts. Count-based correction penalizes measurement richness without addressing a coherent inferential problem.
Multiple primary outcomes	Are several distinct pre-specified constructs each to be tested?	One pre-specified direct test per construct	Not required per construct	Each construct is tested once with a pre-specified primary outcome. Correction across constructs is not required, but the construct list must be justified and all outcomes reported.

## Data Availability

The original contributions presented in the study are included in the article/**Supplementary Material**. Further inquiries can be directed to the corresponding author.

## References

[B1] Anderson T. Post E. G. (2025). Multiplying alpha: When statistical tests compound in sports medicine research. J. Athl Train 60, 548–550. doi: 10.4085/1062-6050-0700.24 40059734 PMC12374235

[B2] Benjamini Y. Hochberg Y. (1995). Controlling the false discovery rate: A practical and powerful approach to multiple testing. J. R. Stat. Soc Ser. B. Stat. Methodol. 57, 289–300. doi: 10.1111/j.2517-6161.1995.tb02031.x 40046247

[B3] Berger J. O. Sellke T. (1987). Testing a point null hypothesis: The irreconcilability of P values and evidence. J. Am. Stat. Assoc. 82, 112–122. doi: 10.1080/01621459.1987.10478397 37339054

[B4] Bland J. M. Altman D. G. (1995). Multiple significance tests: The Bonferroni method. BMJ. 310, 170. doi: 10.1136/bmj.310.6973.170 7833759 PMC2548561

[B5] Goodman S. N. (1999). Toward evidence-based medical statistics. 1: The P value fallacy. Ann. Intern. Med. 130, 995–1004. doi: 10.7326/0003-4819-130-12-199906150-00008 10383371

[B6] Murphy J. Caldwell A. R. Mesquida C. Ladell A. J. M. Encarnación-Martínez A. Tual A. . (2025). Estimating the replicability of sports and exercise science research. Sport Med. 55, 2659–2679. doi: 10.1007/s40279-025-02201-w 40522610 PMC12513899

[B7] Neyman J. Pearson E. S. (1933). IX. On the problem of the most efficient tests of statistical hypotheses. Philos. Trans. R. Soc Lond. Ser. Contain Pap Math. Phys. Char 231, 289–337. doi: 10.1098/rsta.1933.0009 34341189

[B8] Perneger T. V. (1998). What’s wrong with Bonferroni adjustments. BMJ. 316, 1236–1238. doi: 10.1136/bmj.316.7139.1236 9553006 PMC1112991

[B9] Rosenthal R. Rosnow R. L. Rubin D. B. (1999). Contrasts and Effect Sizes in Behavioral Research: A Correlational Approach (Cambridge: Cambridge University Press). doi: 10.1017/CBO9780511804403

[B10] Rothman K. J. (1990). No adjustments are needed for multiple comparisons: Epidemiology. Epidemiology. 1, 43–46. doi: 10.1097/00001648-199001000-00010 2081237

[B11] Rubin M. (2021). When to adjust alpha during multiple testing: A consideration of disjunction, conjunction, and individual testing. Synthese. 199, 10969–11000. doi: 10.1007/s11229-021-03276-4 30311153

[B12] Savitz D. A. Olshan A. F. (1995). Multiple comparisons and related issues in the interpretation of epidemiologic data. Am. J. Epidemiol 142, 904–908. doi: 10.1093/oxfordjournals.aje.a117737 7572970

[B13] Scheffe H. (1953). A method for judging all contrasts in the analysis of variance. Biometrika. 40, 87. doi: 10.2307/2333100 27246801

[B14] Simmons J. P. Nelson L. D. Simonsohn U. (2011). False-positive psychology: Undisclosed flexibility in data collection and analysis allows presenting anything as significant. Psychol. Sci. 22, 1359–1366. doi: 10.1177/0956797611417632 22006061

[B15] Tukey J. W. (1949). Comparing individual means in the analysis of variance. Biometrics. 5, 99. doi: 10.2307/3001913 18151955

